# SWOT analysis to reduce surgical center idleness and increase revenue in a hospital

**DOI:** 10.31744/einstein_journal/2023GS0408

**Published:** 2023-07-25

**Authors:** Kamila da Silva Rola Fachola, Marli de Carvalho Jericó, Ângela Silveira Gagliardo Calil, Danielly Negrão Guassú Nogueira, Fernanda Nayara Senhorini, Renata Prado Bereta Vilela, Paula Buck de Oliveira Ruiz, Patrícia de Carvalho Jericó, Pedro Paulo de Carvalho Jericó

**Affiliations:** 1 Faculdade de Medicina de São José do Rio Preto São José do Rio Preto SP Brazil Faculdade de Medicina de São José do Rio Preto, São José do Rio Preto, SP, Brazil.; 2 Universidade Estadual de Londrina Londrina PR Brazil Universidade Estadual de Londrina, Londrina, PR, Brazil.; 3 Hospital São Lucas Porto Alegre RS Brazil Hospital São Lucas, Porto Alegre, RS, Brazil.

**Keywords:** Health management, Patient safety, Quality of health care, Nursing, Surgicenters, Total quality management, Organization and administration, Costs and cost analysis

## Abstract

**Objective:**

To reduce surgical center idleness by analyzing the competitive structure of the surgical center in a hospital, and thereby generate value in operations and increase hospital revenue.

**Methods:**

The study used qualitative and quantitative methods and an action research approach involving the surgical center leadership of a small private specialized hospital in southeastern Brazil. We used the Strengths, Weaknesses, Opportunities, and Threats or SWOT tool to analyze the competitive structure of the surgical center and then implemented interventions as proposed by the science of improvement method proposed by the Institute of Healthcare Improvement.

**Results:**

By applying the SWOT tool, we identified a concentration of surgeries in the specialty of Otolaryngology and the need to establish a health management system to reduce the idleness of the operating rooms. Based on subsequent intervention, procedures from other specialties were inserted that increased surgical production by 2.62X, reduced idleness by 67.84%, and increased revenue by over US$ 276,609.87 in 2018 compared to the previous year 2017.

**Conclusion:**

Investing in quality, surgical schedule management, and inducting new surgeons to the clinical staff resulted in decreased surgical idleness, increased production, better uniformity in scheduling, and increased revenue, while costs remained below the linear trend, allowing for increased profits.

**Figure:**
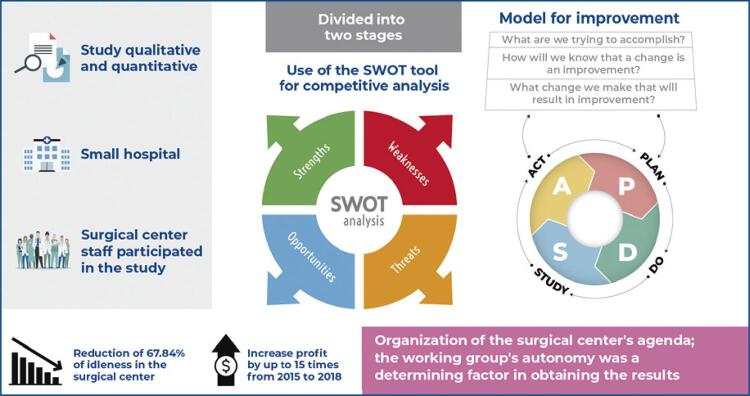

## INTRODUCTION

Over the years, minor or outpatient surgeries have increased in healthcare bringing numerous advantages to patients. The benefits include reduced risk of hospital infection, minimal change in the patient’s routine, quicker return to daily activities, and decreased morbidity and mortality. In addition, hospitals benefit from the greater availability of beds and cost reduction.^( 1 )^

Most hospitals in Brazil are privately managed (63.3%) and small (57.3%), that is, having up to 50 beds. The majority are for-profit (56.9%). When well-managed, surgical centers can account for most of the revenue in small hospitals. Poor management, on the other hand, can result in waste, idleness, and health risks.^( 2 )^

Profits are a constant source of concern for hospitals. Data from the National Association of Private Hospitals (ANAHP - *Associação Nacional de Hospitais Privados* )^( 3 )^ show that the average margin of Earnings Before Interest, Taxes, Depreciation, and Amortization (EBITDA) of hospitals fell from 14.18% in 2016 to 12.40% in 2019, and 89.91% of revenue depended on Private Health Providers (PHPs). Further, in 2018, 330 private hospitals closed in Brazil, most of them small-sized.^( 2 )^

It is crucial to elucidate the relevance of proactive management of the surgical center since it is known that the balance between production capacity and demand is essential for its good performance. This involves a range of factors, from internal processes to its positioning in the market. The cost of a nonfunctioning operating room comprises wasted time, resources, and professionals.^( 4 )^ Therefore, productivity should be monitored using indicators such as occupancy rate, mean duration of surgery, and operating capacity of the operating room to provide a comprehensive analysis of factors that determine gains and losses.^( 5 )^

Managing all the variables related to surgical center dynamics is a considerable challenge. These include the preparation of a well-dimensioned surgical map, the administration of usage intervals of the rooms, the forecast of the availability of instruments, equipment, and materials, and the management of surgical risk, all essential activities for good performance.^( 6 )^

To support organizations in preparing their market strategies and streamline their internal processes, one of the tools that can be used is which stands for Strengths, Weaknesses, Opportunities, and Threats (SWOT). SWOT is effective for strategic analysis and can be carried out in various scenarios to evaluate organizational environments systematically.^( 7 )^

Hospitals require an organizational overhaul that makes processes more patient-centered, while simultaneously increasing productivity and financial returns.^( 8 )^ Clinical outcomes, customer experience, and cost reduction must be optimally balanced. This mission is complex and challenging, especially when involving operating rooms of small-sized hospitals.^( 9 )^

## OBJECTIVE

To analyze the competitive structure of the operating room of a hospital to reduce idleness, generate value in operations, and increase hospital revenues.

## METHODS

This study applied qualitative and quantitative methods to an action research approach. This methodology promoted collaborative development of a process through the construction of knowledge and social change. The premise of action research is dialogue, which is divided into three moments: the first being investigation (collection of information, observation, and survey of characteristics), the second, thematization (critical reflection on the researched facts and their theoretical elaboration), and the last, programming/action (design or planning of programmed actions; execution and evaluation).^( 10 )^

The study was conducted between 2015 and 2018 in Otorhinolaryngology and Surgical Specialties Hospital, a small private hospital in southeastern Brazil. This hospital was opened in 2015 and mainly served the specialty of Otorhinolaryngology, operating Monday to Friday from 7 am to 6 pm and on Saturdays from 7 am to 11 am. It has 19 inpatient beds and four operating rooms, one of which is rented to an ophthalmology hospital; thus only three operating rooms were included in this research.

All the surgical center staff, including the Clinical Director, Director of Anesthesiology, Pharmacy Manager, Hospital Administrator, and Nursing Manager, were invited to participate in this research. The study was divided into two stages: in the first stage, held in March 2017, the participants performed an analysis of the organization’s position before the market using the SWOT tool that allows the examines the competitive structure of an organization from the four perspectives.^( 11 )^

In the second stage of the research, which was conducted from April to December 2017, an intervention was rolled out based on the results corresponding to the four variables, to decrease the weaknesses and threats and strengthen the strengths and opportunities using the science of process improvement recommended by the Institute of Healthcare Improvement.^( 12 )^ The improvement model applied the three critical questions to each proposal: What are we trying to accomplish? How will we know if this change is an improvement? What changes can be made that will result in improvement?

Next, the proposals were submitted to the PDSA12 cycle, also proposed by Institute of Healthcare Improvement. The acronym refers to Plan (P), Do (D), Study (S), and Act (A) with corresponding activities in each step. We also used the brainstorming technique.^( 13 )^

We used boxes to describe qualitative results. Quantitative results were organized in a Microsoft Excel 2010 spreadsheet. Statistical analysis was performed using IBM SPSS Statistics for Windows, Version 23.0 (Armonk, NY: IBM Corp.). Normality was tested using the Kolmogorov-Smirnov test. Continuous data were tested using the Mann-Whitney *U* test when not normally distributed. Spearman rank correlation was used to test non-parametric data.

The occupancy rate and idle rate^( 6 )^ were used to evaluate the operational capacity.

Occupancy rate: effective use of the operational capacity of the Surgical Center, was calculated by the time (in minutes) of full use of the surgical center plus the time spent cleaning and preparing it, divided by the total number of hours that the surgical center was available (7-18 hours = 660 minutes) multiplied by 100; Idle Rate: is the total hours that the Surgical Center was available, calculated by subtracting the Occupancy Rate from 100%.

Thus, the total adequate capacity was the sum of the surgical minutes plus 12 minutes, referring to the time spent cleaning and preparing the surgical center at the institution.

The value of US$ 2,19 per surgical minute was considered to calculate the cost of surgery, which was added to the cost of material and medication. In addition, the hospital’s daily rate was added to the cost, which ranged from US$ 0,00 to US$ 110,31, depending on the patient’s length of stay. The value of the investments made was obtained through the purchase records and hospital administration sector. The price table of private procedures was used to identify revenues, and the values were corroborated with that of the health insurance companies. The revenues from the sales taxes on Orthotics, Prostheses, and Special Materials (OPSM) were not investigated because the same procedures may or may not make use of OPSM, depending on the contract with the private health providers.

To facilitate standardization, the Brazilian real, which was used in the initial calculations, was later converted into US dollars using the Central Bank of Brazil’s average rates/year of R$ 3.3196/US$ 1.00 (2015), R$ 3.4883/US$ 1.00 (2016), R$ 3.1910/US$ 1.00(2017), and R$ 3.6152/US$ 1.00 (2018).

The research was approved by the Ethics Committee of the *Faculdade de Medicina de São José do Rio Preto,* CAAE: 34118620.9.0000.5415; # 4.205.859.

## RESULTS

The results of the SWOT matrix analysis demonstrated the internal and external factors that influenced the hospital activity, and the main points raised are shown in table 1 . The most expressed negative points were the unorganized surgical schedule (Weakness) and the lack of knowledge regarding hospital structure among physicians from other specialties (Threats). Among the positive points, the most impacting were the low rate of infections, hospitality (Strength), and competitive surgical packages (Opportunities).

**Table 1:** Representation of the SWOT analysis results regarding the operating rooms

	Strength	Weakness
Internal factors	- Hospitality management - Low infection rate or no infections - Communicative and accessible anesthesia team - Sedation and anesthesia protocol with few or no postoperative adverse events, such as nausea and vomiting - Arsenal of diversified surgical instruments that can meet the various types of surgery - Incudes a materials center	- Non-availability of an ICU - There is no management of the surgical agenda based on supply *versus* demand and demand variability - No defined quality/patient safety actions and processes - Lack of the nursing staff training - Insufficient number of nursing professionals - Operating room three lacks safety structures - Concentration of a single medical specialty
	**Opportunities**	**Threats**
External factors	- Competitive surgical bundles/packages - Location and accessibility - A reputed hospital in the city in the area of Otorhinolaryngology - Small hospital that facilitates the interface between surgeons and senior management	- Surgeons think that the hospital serves only the specialty of Otorhinolaryngology - Surgeons do not know the hospital - Surgeons with attachment/familiarity with other institutions - Large number of competing hospitals

ICU: intensive care unit.

Based on the weaknesses and threats found in the SWOT matrix, a survey was conducted on the main points could be improved to no longer be negative areas. Table 2 displays the action plan for each topic raised and the annual investment identified to try to solve the problem. The investment made in the year 2017 was 1.86X higher than the one made available in 2018 since the measures implemented in 2017 were maintained and improved for the year 2018.

**Table 2:** Action plan and an annual investment of the main points observed in the SWOT matrix for the weaknesses and threats in 2017 and 2018

Dimension	SWOT result	Main action plan points	Yearly investment	

			2017	2018
Weakness	Unavailability of ICU	Sign ICU contract with large hospital and mobile ICU	US$ 940.14	US$ 829.83
	There is no management of the surgical schedule around supply *versus* demand and variability of demand	Establish fixed days and times for surgeons Prioritize longer surgeries in the first schedule Concentrate the otorhinolaryngology surgeries of the same doctor in a single period Schedule minor surgeries in the afternoon	Hours worked	Hours worked
	No defined quality/patient safety actions and processes	Carry out process mapping and risk management to improve internal processes	US$ 11,142.90	US$ 9,835.42
	Lack of the nursing staff training	Conduct staff training based on the design of internal quality and patient safety processes, prioritizing realistic simulation training	Hours worked	Hours worked
	Insufficient number of nursing professionals	Perform personnel downsizing for the nursing staff Hiring of one nurse and two Licensed Practical Nurses Paid Internship Program in Nursing	US$ 45,126.92 US$ 6,769.04	US$ 39,831.82 US$ 5,974.77
	Operating room three without a secure structure	Purchase surgical lipoaspirator, surgical table, surgical focus, and video endoscope	US$ 10,968.35 operating table US$ 37,605.77 video endoscope; US$ 1,316.20 surgical lipoaspirator US$ 4,901.28 surgical roof focus	US$ 0.00
Threat	Surgeons think that the hospital serves only the specialty of Otorhinolaryngology	Change the logo to specialty hospital	US$ 470.07	US$ 0.00
	Surgeons do not know the hospital	Active search for surgeons through office visits promoting the strengths and opportunities	US$ 156.69	US$ 0.00
	Surgeons with attachment/familiarity with other institutions	First trial program where the surgeon is invited for a surgical experience without being part of the clinical staff	No investment	No investment
	Large number of competing hospitals		No investment	No investment
Total			US$ 119,397.37	US$ 56,471.84

ICU: intensive care unit.

Over the years and with the interventions performed in the first quarter of 2017, we identified a 2.62-fold increase in surgeries from 2015 to 2018 (n=8,809 surgeries).

The negative points presented were related to the unorganized surgical schedule and the lack of knowledge of the hospital structure among physicians from other specialties. Interventions were conducted to organize the surgical schedule and visitations were made to doctors to inform them of the procedures performed in this hospital. The idleness rates were 60.48% in 2015, 53.55% in 2016, 37.67% in 2017 and 19.45% in 2018. Through these activities, it was possible to reduce the idleness of the surgical center by 67.84%, representing a 3.10-fold reduction.

Spearman’s test found a positive association between the time (years) and the number of surgeries (p=0.013; R=0.98). The more time passed, the greater the number of surgeries, showing that the interventions impacted the increase in surgical production.

Furthermore, after organizing surgery schedules according to the types, it was possible to equalize the demand for surgeries across periods, without concentrating all surgeries only in the morning. In 2015, surgeries were performed exclusively in the morning, while in 2018, approximately 60% of surgeries were in the morning and 40% in the afternoon.

**Figure 1:**
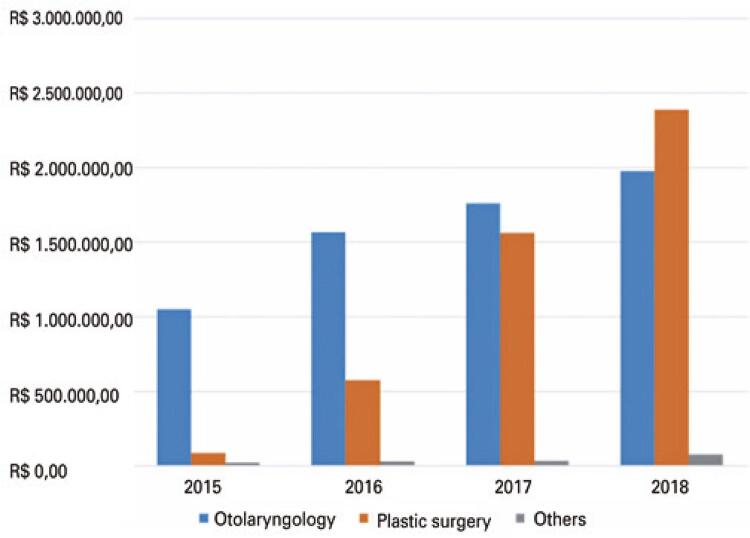
Surgery revenue by specialty (Otolaryngology, Plastic Surgery, and Other) from 2015 to 2018

The increase in hospital revenue was one of the main objectives met; from 2015 to 2018, there was an increase of 9.61 times in revenue. The results showed that although the Plastic Surgery specialty represented 20.83% of the surgeries, it contributed 41.43% of the revenue. This uptake, specifically of plastic surgeons, can be seen in figure 1 .

The primary sources of revenue came from health insurance providers (75.13%), with medical cooperatives being the largest payers in terms of the number of procedures (38.29%), followed by self-management plans (35.38%). Although predominant, the operators made the lowest payments per procedure, with 53.20% of the procedures paid for by medical cooperatives involving amounts up to R$ 1,000.00 (US$ 313.38), while 98.18% of the private payments were above R$ 3.000.00 (US$ 940.14) per procedure, as shown in table 3 . Private payments grew by 121.53% from 2015 to 2018.

**Table 3:** Distribution of revenues by type and payment amount (US$)

Variables NASH Classification	Up to 313.38 n (%)	313.39 to 626.76 n (%)	626.77 to 940.14 n (%)	>940.14 n (%)	Total n (%)
Group Medicine	6 (0.15)	3 (0.09)	1 (0.11)	0 (0.00)	10 (0.11)
Practitioner cooperatives	2,181 (53.20)	1,111 (32.84)	79 (8.40)	2 (0.52)	3,373 (38.29)
Own/self-management plans	1,735 (42.32)	1,265 (7.39)	117 (12.43)	0 (0.00)	3,117 (35.38)
Group Health Insurance	0 (0.00)	3 (0.09)	1 (0.11)	0 (0.00)	4 (0.05)
Health insurance + private	16 (0.39)	77 (2.28)	12 (1.28)	5 (1.30)	110 (1.25)
Private	147 (3.59)	899 (26.57)	731 (77.68)	378 (98.18)	2,155 (24.46)
Hiorp social^#^	11 (0.27)	25 (0.74)	0 (0.00)	0 (0.00)	36 (0.41)
Courtesy	4 (0.10)	0 (0.00)	0 (0.00)	0 (0.00)	4 (0.05)
Total	4,100 (100.00)	3,383 (100.00)	941 (100.00)	385 (100.00)	8,809 (100.00)

^#^ Hiorp social was an exclusive agreement created to assist low-income people with affordable prices that only covered costs and did not bring profits to the hospital.

NASH: National Agency of Supplementary Health.

It was possible to increase profit by up to 15 times from 2015 to 2018, with all the interventions. The revenue of R$ 1,158,116.78 (US$ 348,872.39) in 2015 rose to R$ 4,446,125.88 (US$ 1,229,842.30) in 2018, and the cost from R$ 1,100,096.84 (US$ 331,394.40) in 2015 to R$ 3,572,499.14 (US$ 988,188.52) in 2018. There was a linear increase in revenue and margin, and the cost was reduced over the years, allowing for an increase in profits.

## DISCUSSION

SWOT analysis is a simple but efficient management tool to evaluate the strategic positioning of an organization. Although some authors do not recommend its application in the healthcare sector,^( 14 )^ considering it too simplistic, the tool has been widely used and represents a competitive advantage.^( 15 )^ The results found in this research showed that the SWOT analysis painted a panorama of the surgical center concerning the competitive market and provided an assessment of internal aspects, allowing the management team to develop more proactive strategies in conducting business.

Among the strengths and opportunities identified, the low infection rate stood out. Undoubtedly, a place with standard or no infection is attractive to both surgeon and patient. It is worth mentioning that this hospital did not systematically evaluate the cases of infection, and only started during this research period. Therefore, this information could have been underreported. The leading causes of infection in hospital environments are the environmental, personnel, equipment, material, vehicles, professional malpractice, and incorrect use of antibiotics.^( 16 )^

Among the weaknesses and threats detected were the insufficient number of nursing professionals and their lack of training. The interventions implemented, such as the correct dimensioning of the nursing team and training, aim to prepare professionals to provide care that improves patient safety, reduces care-related complications, increases patient and physician satisfaction, and rationalizes costs.^( 17 )^ When it comes to nursing dimensioning, the Federal Council of Nursing (COFEN) establishes that the number of professionals in the surgical center should consider the hours of assistance according to the scale of the surgery, the cleaning time of the rooms, and the waiting time for surgeries. According to the scale of the surgery, it is recommended that there be at least one nurse for three operating rooms, one circulator, and one operator per room.^( 18 )^

Patient safety actions were suggested to address another weakness. A study demonstrated that the implementation of the safe surgery checklist significantly increased the chance of the patient receiving adequate treatment, reducing complications, and mortality, improving teamwork, and reducing costs.^( 1 , 19 )^

Risk management activities, such as pre-anesthetic consultation, allowed the anesthesiologist to evaluate the patient regarding risk and suitability to the institution’s profile, that is, only patients with ASA I and II risks were acceptable. Medication reconciliation and prescription screening by the pharmacist, contracts with blood banks, and backup intensive care unit were also essential for patient safety.

Actions pertaining to the surgical planning management were also executed, and the rules for the surgical map elaboration were drastically altered. It went from scheduling according to availability to specific days for each surgeon and prioritizing the longer surgeries in the morning. Consequently, the smaller surgeries were scheduled in the afternoon. This change balanced the surgical map, eliminating the concentration of surgeries in the morning from 80% to 60%. The balance in the scheduling of surgeries not only looked at distribution to fill the hours but focused on patient safety. The significant surgeries prioritized in the morning had the objective of managing the risk of complications in the immediate postoperative period. If the patient needed some intervention, the probability was that it would still occur in the afternoon and not during the night when fewer staff were available.

The surgical scheduling started to be carried out with a forecast of the equipment, surgical instruments, and beds available. In the surgical room that only performed minor procedures, investments were made in a surgical table and a ceiling focus to improve the structure, totaling R$ 50,640.00 (US$ 15,869.63). It was possible to perform medium and large procedures in this room, especially plastic surgery that require different bends and rotations of the tables. A new video endoscope was acquired so that Otorhinolaryngology surgeries could be performed simultaneously in different rooms. Besides this, a rotation of the surgeries that followed without using the video endoscope allowed greater productivity in the specialty. As for the surgical instruments, we encouraged the surgeons themselves to acquire them so that more surgeries could be scheduled in a row. Thus, the hospital did not need to invest in new instruments.

The dynamics of the surgical center allowed high productivity, but there was no increase in the number of beds. Thus, the secretary who organized the surgical chart started to make the appointments with forecasts of available beds, which could vary from 10 to 19 beds, depending on the type of admission, with private or shared quarters, or if the surgery would require an overnight stay.

Given the complexity of the surgical map organization, as a risk management activity, it was incorporated into the nurses’ duties to check the schedule a fortnight, week, and the day before the procedure, wherein they would check the necessary equipment and instruments, available beds and had the autonomy to block the agenda, contact surgeons and make any changes that were necessary for the safety of the surgical procedure. Concomitantly, the pharmacy professionals performed the same check concerning materials, medications, and OPSM.

A prior study indicated that a surgical map is a fundamental tool for planning. It is possible to allocate the rooms, times, and procedures and brings essential records regarding the scale of the surgery, special equipment, and teams. In this study, the map improved the distribution of surgeries by increasing the number of procedures without increasing the operating rooms.^( 20 )^

Alterations to the surgical map was the biggest challenge, as it involved several players, such as the hospital directors, anesthesiologists, the nursing team, and already loyal surgeons who had preferences for the morning schedule. To manage the transition, the nurse had to play a political role in the transformation process and communicate a win-win process. The surgeons who were transferred to the afternoon period were allowed to have consecutive surgeries, organized with pre-booking of room and time, making it easier to conciliate office and personal life. Thus, if there were no appointments up to seven days before the surgical procedure, the room would be released.

One of the sensitive issues to manage was the question of privileges, especially with the Otolaryngology team who, before the intervention, were the dominant specialty, while other specialties behaved like guests. After the changes, everyone became a member of a single clinical staff with pre-established rules, and some even verbalized the feeling of loss of privileges. Therefore, the upper management needed to follow the same established rules for this specialty. As far as possible, requests such as preferences for a particular operator or circulator and changes to morning hours in case of availability were accommodated.

In this context, nurses must exercise their political and managerial role, which means having the ability to interact with other groups, resolve conflicts, and lead a cooperative action toward a common goal.^( 21 )^

The identification of the surgical profile showed that otorhinolaryngology surgeries continue to be the most frequent but that plastic surgeries increased approximately 30% in the period evaluated. A study carried out in a public university hospital, with 12,114 surgical procedures, presented the Orthopedics specialty as the most frequent, with Plastic Surgery and Otorhinolaryngology as the fifth and sixth specialties, respectively. This same study showed that identifying the average times of the procedures helps in the planning of the surgical center and contributes to reducing delays, optimizing the unit and hospital organization.^( 22 )^

Moreover, optimizing the surgical time can contribute to the quality of the hospital. This study showed that the longer the surgical time, the longer the length of hospital stay, and the mean surgical time was between 72 and 102 minutes. Another study^( 22 )^ found that the operative time for plastic surgery averaged 158 minutes, and for otorhinolaryngology, approximately 129 minutes.

Among the results obtained with the interventions, the reduction of surgical idleness was significant, going from about 60% to 19%. A prior study demonstrated idleness of 20.61% in a private tertiary hospital with good optimization.^( 6 )^ In contrast, another study showed that 43.40% idleness requires increased productivity to reach the break-even point, making potential gains possible.^( 23 )^ Idleness is an invisible waste and investing in management and change is more effective than building new operating rooms.^( 5 )^

The increase in plastic surgeries increased direct and private payments to the hospital since 98.18% of the surgeries with values greater than R$ 3,000.00 (US$ 940.14) were private. Comparing the data obtained in this research with those from ANAHP,^( 3 )^ it was possible to observe that private revenues were higher (24.46%) more than comparable (3.26%). Private prescriptions are beneficial to the hospital because they are freely negotiated, with higher prices per procedure, free of disallowances, and fast payment. On average, 3.41% of the prescriptions coming from private health providers are disallowed, and the average time for receipt is 66.95 days. In this study, 38.29% comes from medical cooperatives, 35.38% from self-management, while in ANAHP the figures are 31.82% and 27.86%, respectively.^( 3 )^

The revenue and overall cost data showed efficiency in the management of the operating room. While revenues grew, costs did not follow the same trend, with profits increasing over the years. A study in the United States showed that investing in management and patient safety can reduce costs by 1,500 dollars per patient. However, investments are necessary, as seen in this research, and the gains can reach 1.1 million dollars per hospital.^( 19 )^

In Brazil, a study of medium-sized hospitals showed that the higher the adoption of practices for good performance, the higher the occupancy rate, and the effectiveness of management decisions and appropriate use of management tools collaborate to improve quality and competitiveness among health organizations.^( 24 )^

All the interventions were possible with the autonomy given by the owner-partners to the strategic group. Monthly strategic meetings that involved presentation of key indicators, enabled a trusting relationship between the top leadership and the group leader’s Nursing Manager. This two-way propensity, willingness to give autonomy while the other aims to gain independence and live up to the trust granted, can be demonstrated in a scenario where there were investments of R$ 380,997.00 (US$ 119,397.37) in 2017, whereas in the previous year, 2016, the hospital was still making a profit of R$ 107,502.09 (US$ 30,817.90). As for the operational team, no resistance was observed to the proposed changes since the employees suggested many of the interventions, facilitating the team’s engagement in an ongoing cultural transformation process.

Finally, a last unscheduled intervention was carried out in mid-April of the same year, when a public hospital in the city closed, and the Otorhinolaryngology residents started performing their activities at the research hospital. These residents’ low demand for surgeries impacted the learning process since they could only help the teaching surgeons. Thus, we created a social program where consultations and surgical procedures were offered to the low-income population at a cost price, with the residents doing them under the supervision of a teacher. During the research period, 36 procedures were performed.

Regarding the limitations of this research, it was not possible to obtain data on delays and cancellations of surgery. In the literature consulted, it was impossible to find studies carried out in hospitals of the same size and profile with which to better compare the results.

## CONCLUSION

We concluded that identifying the positive and negative points in the management of small hospital is essential to determine the best ways to intervene. Our findings also showed that plastic surgery brings a vital increase to hospital revenue. Besides the organization of the surgical center’s agenda, the nurses’ training and autonomy reduce the idleness rate and increase efficient use of the hospital. With more autonomy and profit-sharing, the increase in private surgeries also stands out. The quality aspects, risk management, and patient safety are pillars of internal development and collaboration both for internal processes and for the competitiveness of the hospital. The working group’s autonomy was a determining factor in obtaining the results.
